# The effect of postoperative wound infections on functional outcome following intra-articular calcaneal fractures

**DOI:** 10.1007/s00402-015-2219-5

**Published:** 2015-04-26

**Authors:** Manouk Backes, Niels W. L. Schep, Jan S. K. Luitse, J. Carel Goslings, Tim Schepers

**Affiliations:** Trauma Unit, Department of Surgery, Academic Medical Center, Meibergdreef 9, PO Box 22660, 1100 DD Amsterdam, The Netherlands

**Keywords:** Calcaneus, Open reduction internal fixation, Extended lateral approach, Postoperative wound infection, POWI, Outcome, AOFAS, FFI, Quality of life

## Abstract

**Introduction:**

High rates of postoperative wound infections (POWI) are reported following the operative treatment of calcaneal fractures. This leads to additional therapy, prolonged hospital stay, burden for patients and increased costs. The primary aim of this study is to evaluate the effect of POWI following the extended lateral approach of displaced intra-articular calcaneal fractures on functional outcome. Secondary aims are assessment of health-related quality of life and patient satisfaction.

**Patients and methods:**

All consecutive adult patients with a calcaneal fracture treated between 2000 and 2011 with open reduction and internal fixation through an extended lateral approach were retrospectively included and sent a questionnaire. Functional outcome was measured using the Foot Function Index (FFI, best score 0 points) and the American Orthopaedic Foot and Ankle Society (AOFAS, best score 100 points) hindfoot score. The EuroQOL-5D was used for quality of life (QOL) and a Visual Analogue Scale (VAS, best score 10 points) for overall patients satisfaction.

**Results:**

Of 135 eligible patients, 94 returned the questionnaire (response rate 70 %). The median FFI was 12 points (IQR 3–33) and AOFAS 79 points (IQR 61–90). The FFI and AOFAS were, respectively, 17 and 9 points higher in favour of patients without POWI (*n* = 69) compared to patients with POWI (*n* = 25). Albeit large differences, they were not statistically significant given the current number of patients. Patients without POWI scored better on all health-related aspects of QOL in the EQ-5D, but this did not reach statistical significance. However, the VAS on overall patient satisfaction did show a statistically significant difference of 1.3 points (9.0 vs 7.7; *p* = 0.01) in favour of patients without POWI. Importantly, a clinically relevant difference was found with the FFI as the estimated minimal clinical important difference of the FFI is 10 points.

**Conclusion:**

Our results implicate that postoperative wound infection leads lower functional outcome scores following calcaneal fracture surgery, but no statistical significance was reached. In addition, patients do not report significant worse QOL or physical impairment. Overall patient satisfaction measured by a VAS was significantly lower in case of a POWI, reflecting the burden caused by a wound complication.

## Introduction

In the last two decades, several studies showed improved outcome following operative treatment in patients with displaced intra-articular calcaneal fractures compared with non-operative treatment [1–5]. Moreover, initial operative management has proven better long-term functional results in case a secondary arthrodesis is required [6].

However, open reduction and internal plate fixation of the calcaneus through an extended lateral approach is infamous for its high rate of postoperative wound infections (POWI) and various risk factors have been identified [7–11]. Wound complications can be divided into minor complications (superficial infection, dehiscence, wound edge necrosis) and major complications (deep infection, osteomyelitis, plate fistula) [12]. Rates of minor and major complications reported in literature are, respectively, 0–21.4 and 0–14.3 % [7, 9].

Costs in patients with a displaced intra-articular calcaneal fracture and a postoperative complication (including wound, implant and neurologic complications, thromboembolism and compartment syndrome) are approximately $2000 higher compared to patients without a complication. In addition, costs in patients requiring a secondary fusion can be up to $74.000 higher [13].

Besides additional medical costs and lengthened hospital stay, a postoperative wound complication leads to an increased burden for the patient [14]. One previous study on wound complications specifically did not find a negative relation between wound complications and outcome; however, this study was hampered by a relatively small number of patients and mainly superficial wound complications [14]. Therefore, little information is available on the effect of POWI on long-term functional outcome.

The primary aim of the current study was to evaluate the effect of POWI following calcaneal fracture surgery on functional outcome. The secondary aims were measuring the effect of POWI on health-related quality of life and overall patient satisfaction.

## Patients and methods

We conducted a retrospective cohort study. All consecutive adult patients over an eleven-year period (January 1st, 2000 to December 31st, 2010) with open reduction and internal fixation of a closed displaced intra-articular calcaneal fracture through an extended lateral approach were assessed for eligibility. Exclusion criteria were patients with a primary arthrodesis, a different surgical approach and inability to fill in a questionnaire (unknown address, not attending outpatient department visits, death or imprisonment).

### Clinical data

Patient characteristics obtained from the electronic hospital’s medical charts were gender, age at trauma and past medical history such as diabetes and nicotine abuse. Trauma characteristics included injured side and trauma mechanism, further subdivided into fall from height or stairs, motor vehicle accident or other. All fractures were classified according to the Essex-Lopresti and Sanders classifications. Initial Böhlers angle was measured by a trauma surgeon specialized in foot and ankle trauma [15, 16].

All patients were seen within 30 days postoperatively by an attending physician. Postoperative wound infections were subdivided in superficial or deep infections by applying the criteria of the US Center for Disease Control and Prevention [12]. A superficial POWI was defined as a wound with signs of infection (confirmed by a positive culture) amendable for conservative treatment with antibiotics. A deep wound infection was confirmed with a positive culture, osteomyelitis, infected hardware or a plate fistula in need for hardware removal, (readmission with) intravenous antibiotics or wound debridement with or without local antibiotic treatment with gentamicin beads or vacuum assisted closure.

Finally, secondary intervention such as implant removal, secondary arthrodesis and number of additional surgical procedures following the initial procedure were registered.

Primary outcome was functional outcome as measured by two area specific outcome scores. Functional outcome was measured using the Foot Function Index (FFI, best score 0 points) [18], and the American Orthopaedic Foot and Ankle Society hindfoot score (AOFAS, best score 100 points) [19]. The AOFAS score was divided into groups according to the literature: a score of 90–100 was graded as an excellent result; 75–89 as good; 50–74 as fair, and less than 49 points was graded as a failure or poor outcome. Both outcome measurements are frequently used in foot and ankle research [17]. Range of motion and alignment was documented for all patients at their final visit to the outpatient clinic in follow-up and these data were obtained from the outpatient medical charts. From the literature, it is known that little additional improvement in the AOFAS score can be expected after 1.5 years of follow-up [18].

Secondary outcome was quality of life (QOL), which was measured by the EuroQol-5D (EQ-5D) [23]. This included assessment of perceived general health on a Visual Analogue Scale (VAS) of zero to 100, in which 100 represented excellent general health (EQ-VAS). In addition, a ten-point VAS, with zero implying maximum dissatisfaction and ten full satisfaction was used to measure patient satisfaction with overall outcome [21].

In addition, questions were asked on ability to work and type of employment; classified as either heavy physical labour or light physical labour. Finally, patients were asked to report on time to return to work and occupational adjustments as a result of their calcaneal fracture.

### Surgical procedure

Open reduction and internal fixation was achieved via an extended lateral approach [9, 15, 24] with a stainless steel 3.5-mm non-locking AO calcaneal plate and screws. A ‘no touch’ technique was applied with K-wires in the talus and cuboid and a tourniquet was rarely used. The goal of surgery was restoration of articular surfaces, calcaneal height, width, length, and correction of varus. Postoperatively a standard pressure bandage was applied. Patients were kept non-weight bearing for a period of 12 weeks and instructed for active range of motion exercises.

### Statistical analyses

Data were analysed using Statistical Package for the Social Sciences (SPSS) version 17.0 (SPSS, Chicago, Illinois, USA). Normality of continuous data was tested with the Kolmogorov–Smirnov test and by inspecting the frequency distributions. Descriptive analysis was performed to compare baseline characteristics between patients with and without POWI. For continuous data, the mean and standard deviation (SD) (parametric data) or medians and interquartile ranges (non-parametric) data were calculated. Differences were assessed using the Student’s *T* test (parametric data) or the Mann–Whitney *U*-test (non-parametric data). Categorical data were compared using the *χ*^2^ test. Finally, the relation between functional outcome and type of fracture, timing of intervention and age was assessed. Also, the relation between POWI and functional outcome/QOL was assessed and corrected for the confounders fracture type and secondary interventions by means of multivariate logistic regression. A *p* value of <0.05 was taken as the threshold of statistical significance. All *p* values are two tailed.

## Results

### Demographics

During the 11-year study period, a total of 182 patients with 195 fractures were treated surgically with open reduction internal fixation through an extended lateral approach. A total of 135 of these patients were included in the study and sent a questionnaire. Patients were excluded because of an unknown address (*n* = 19), not attending the outpatient department (*n* = 17), primary arthrodesis (*n* = 5), death (*n* = 4) or imprisonment (*n* = 2). A total of 94 patients returned the questionnaire, resulting in a 70 % response rate with a median follow-up of almost 6 years (71 months). Of these, 25 patients suffered from a POWI, of which 12 patients had a deep POWI.

Patient characteristics and secondary interventions of both the responding patients and non-responding patients are displayed in Table 1. Patients not responding were more frequently male, smokers and younger of age (*p* < 0.05). In univariate analysis, no association was found between the occurrence of POWI and male gender (*p* < 0.344), younger age (0.854) or nicotine abuse (0.826). The median follow-up was 33 months following open reduction and internal fixation.

**Table 1:** Patient, trauma, fracture, surgical characteristics and secondary intervention of respondents and non-respondents following intra-articular calcaneal fracture surgery

	Patients with response (*N* = 94)	Patients without response (*N* = 41)	*p* value
Patient characteristics			
Male (%)	57 (61)	32 (78)	<0.05
Median age in years at time of trauma (range)	48 (14 to 75)	44 (12 to 68)	<0.05
Diabetes mellitus (%)	8 (8)	3 (7)	NS
Nicotine abuse (%)	33 (35)	24 (59)	<0.05
Median follow-up in months (range)	71 (26 to 157)	NA	NA
Trauma characteristics			
Unilateral (%)	81 (86)	41 (100)	<0.05
Traumamechanism (%)			NS
Fall from height or stairs	81 (86)	38 (93)	
MVA	6 (6)	1 (2)	
Other	7 (7)	2 5)	
Fracture characteristics			
Concomitant foot/ankle injury (%)	15 (16)	2 (5)	NS
Essex-Lopresti classification (%)			<0.05
Joint depression type	37 (39)	27 (66)	
Tongue type	49 (52)	9 (22)	
Combined type	1 (1)	1 (2)	
Unknown	6 (6)	4 (10)	
Sanders classification (%)			NS
I + II	64 (68)	30 (73)	
III + IV	23 (24)	8 (20)	
Unknown	7 (7)	3 (7)	
Median pre-operative Böhlers angle in degrees (range)	6.8 (−7.5 to 15.2)	8.2 (−0.2 to 8.2)	NS
Surgical characteristics			
Wound complications (%)	29	9	NS
Minor			NS
Wound dehiscence	4 (4)	2 (5)	
Superficial POWI with oral antibiotics	13 (16)	2 (5)	
Major			NS
Deep POWI with iv antibiotics/surgical debridement	5 (5)	5 (12)	
Deep POWI with implant removal	7 (7)	–	
Secondary intervention			
Implant removal (%)	50 (53)	15 (37)	NS
Secondary arthrodesis (%)	9 (10)	2 (5)	NS
Median number of surgeries including implant removal (range)	2 (1 to 9)	1 (1 to 5)	NS

*POWI* postoperative wound infection, *NA* not available, *NS* not significant, *MVA* motor vehicle accident

### Functional outcome and quality of life

Primary and secondary outcomes are presented in the first row of Table 2. Patients with superficial or deep POWI showed a difference in FFI (26−9 = 17 points) and AOFAS (81−72 = 9 points) compared to patients without POWI. However, these differences were not statistically significant given the current number of patients. According to the AOFAS score, a good to excellent result was reached in 54 % of patients (62 % in group without POWI and 32 % in group with POWI). A POWI occurred significantly less often in the group with good or excellent outcome and more often in the group with poor or fair outcome (*p* = 0.017).

**Table 2:** Functional outcome and quality of life measurements in patients with or without a postoperative wound infection following intra-articular calcaneal fracture surgery

Patients (*N*)	FFI (SD)	AOFAS (SD)	EQ-5D index (SD)	EQ-VAS (SD)	VAS (SD)
All (94)	12 (20)	79 (21)	0.83 (0.11)	80 (15)	8.7 (2.1)
No POWI (69)	9 (20)	81 (22)	0.86 (0.11)	78 (13)	9.0 (1.6)
POWI (25)	26 (19)	72 (17)	0.81 (0.12)	80 (19)	7.7 (2.9)*
Deep POWI (12)	23 (21)	72 (17)	0.82 (0.13)	78 (25)	7.6 (3.5)**

*SD* standard deviation, *N* number, *POWI* postoperative wound infection, *FFI* Foot Function Index, *AOFAS* American Orthopaedic Foot and Ankle Society, *EQ*-*5D* EuroQol-5D, *VAS* Visual Analogue Scale on patient satisfaction

* *p* = 0.01 comparing patients with a PWI to patients without a POWI

** *p* = 0.03 comparing patients with a deep PWI to patients without a POWI

**Fig. 1:**
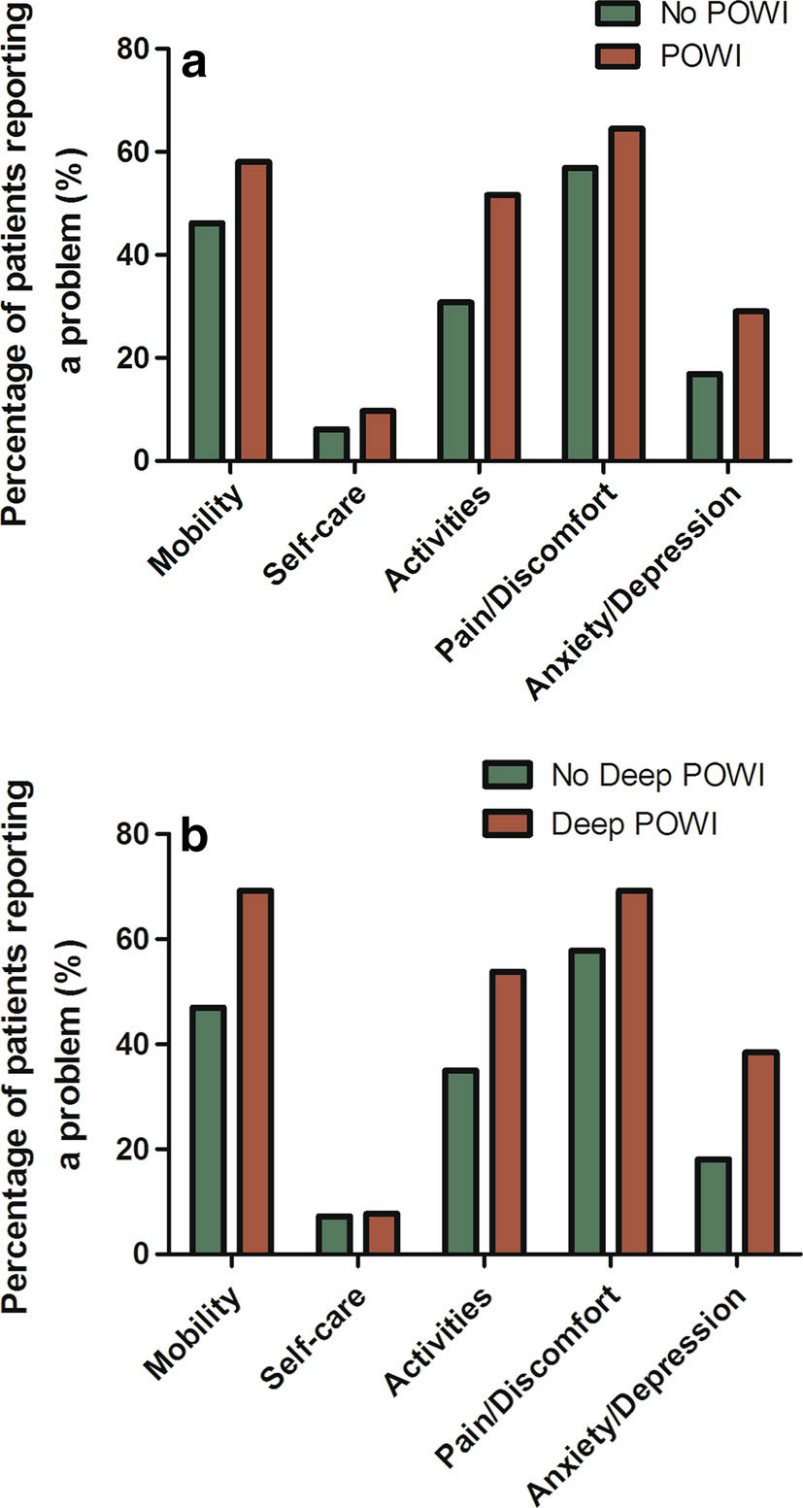
**a** Percentage of patients with a postoperative wound infection versus patients without a postoperative wound infection reporting a problem in the EQ-5D (not significant). **b** Percentage of patients with a deep postoperative wound infection versus patients without a postoperative wound infection reporting a problem in the EQ-5D (not significant). *POWI* postoperative wound infection, *EQ*-*5D* EuroQol-5D

On the other hand, the VAS on overall patient satisfaction did show a significant difference of 1.3 points (9.0 vs 7.7; *p* = 0.01) in favour of patients without POWI (Table 2). When looking at the QOL measurements and the percentage of patients reporting a problem, patients without (deep) POWI scored better on all health-related aspects of QOL in the EQ-5D (Fig. 1). However, this did not reach statistical significance.

Median time to return to work following calcaneal fracture treatment was 4 months (IQR 2–9). Return to work was 6.5 months (IQR 3.5–9.5) in patients with heavy physical labour and 3.5 months (IQR 2–9) in patients without heavy physical labour. In patients performing heavy or light physical labour, the occurrence of POWI, return to work following trauma, adjustment of work environment and inability to work following fracture treatment were not statistically different (*p* > 0.05). Seventeen percent of patients (*n* = 16) was not able to return to their previous work and another 31 % (*n* = 33) required adaptations regarding work environment. In addition, no significant association was found in physical impairment between patients with and without POWI (*p* > 0.05) (Table 3).

**Table 3:** Patient reports on physical impairment prior to and following closed calcaneal fracture surgery

Parameter (*N* of responders)	Pre-trauma (%)	Post-trauma (%)	No POWI, *N* = 69 (%)	POWI, *N* = 25 (%)	*p* value
Practicing sports (91)	49 (52)	37 (40)	27 (39)	10 (40)	0.81
Running (89)	89 (95)	44 (47)	37 (54)	7 (28)	0.09
Ankle stiffness (90)					
Never		23 (24)	20 (29)	3 (12)	0.17^#^
In morning		42 (45)	33 (48)	9 (36)	
Always		25 (27)	14 (20)	11 (44)	
Walk on bare foot (91)					
Easily		57 (61)	45 (65)	12 (48)	0.32^##^
Experience difficulties		28 (30)	19 (28)	9 (36)	
Not possible		6 (6)	4 (6)	2 (8)	
Shoe wear (94)					
Normal footwear		64 (68)	45 (65)	19 (76)	0.45^###^
Orthopaedic adjustments		18 (19)	13 (19)	5 (20)	
Orthopaedic shoes		12 (13)	11 (16)	1 (4)	

*N* number, *POWI* postoperative wound infection

^#^No ankle stiffness compared to stiffness and morning stiffness (*χ* ^2^)

^##^Easily compared to experiencing difficulties and not possible (*χ* ^2^)

^###^Normal footwear compared to orthopaedic footwear (*χ* ^2^)

### Secondary interventions

In 50 patients (52.1 %), implants were removed (vs 37 % in non-responding patients). In seven patients, this was the result of ongoing infection following initial fracture surgery and in four patients because of a fistula or infection after more than 30 days. In nine patients (9.6 %), a secondary arthrodesis of the posterior talocalcaneal (PTC) joint was deemed necessary, of which three patients suffered from a deep and one from a superficial POWI following the initial procedure. Need for implant removal and secondary arthrodesis was both associated with the occurrence of POWI (*p* < 0.05).

Patients in which implants were removed scored significantly better on the FFI (*p* = 0.023) compared to patients without implant removal with a median of 17 vs 8. The AOFAS (73 vs 79), QOL measurements EQ-5D (0.83 vs 0.87), EQ-VAS (75 vs 80) and VAS of overall treatment (8.0 vs 9.0) showed no significant difference.

Patients with secondary PTC arthrodesis did score significantly worse than patients without secondary arthrodesis on FFI (44 vs 9, *p* < 0.001), AOFAS (57 vs 81, *p* = 0.001), QOL EQ-5D Index (0.71 vs 0.86, *p* < 0.001) and VAS for satisfaction (6.5 vs 9.0, *p* = 0.001).

### Multivariate regression analysis

No statistical significance was found between the AOFAS score and surgery within 1 or 2 weeks following trauma (respectively, 0.517 and 0.186) and neither for the FFI (respectively, 0.586 and 0.146). Therefore, no association was found between timing of intervention and functional outcome. The median age of the responding patients was 48 years and no differences were found in functional outcome between patients older or younger than 48 years and their AOFAS or FFI. Also, no association was found between the type of fracture using the Sanders classification and functional outcome/QOL (Table 4).

**Table 4:** Functional outcome and quality of life measurements in patients with or without a postoperative wound infection per Sanders classification

Patients (*N*)	FFI (*p*)	AOFAS (*p*)	EQ-5D index (*p*)	EQ-VAS (*p*)	VAS (*p*)
Sanders 1 (8)	0.456	0.763	0.169	0.099	0.153
POWI (3)					
No POWI (5)					
Sanders 2 (55)	0.263	0.553	0.743	0.467	0.144
POWI (15)					
No POWI (40)					
Sanders 3 (21)	0.238	0.133	0.296	0.587	0.088
POWI (5)					
No POWI (15)					
Sanders 4 (2)	NA	NA	NA	NA	NA
POWI (2)					
No POWI (0)					

*N* number, *POWI* postoperative wound infection, *FFI* Foot Function Index, *AOFAS* American Orthopaedic Foot and Ankle Society, *EQ*-*5D* EuroQol-5D, *VAS* Visual Analogue Scale on patient satisfaction

Multivariate analysis was performed to assess the relation between POWI and functional outcome/QOL corrected for the confounders fracture type and secondary interventions. No association was found for functional outcome. However, patient satisfaction on overall treatment remained significantly higher in patients without POWI (*p* = 0.008).

## Discussion

No statistically significant association was found with postoperative wound infection on functional outcome following calcaneal fracture surgery. However, patients with a POWI reported a poor and fair outcome significantly more often compared to patients without a POWI. Unfortunately, the minimal clinically important difference (MCID) is unknown for both the AOFAS and the FFI [25]. However, the estimated MCID can be calculated as one half of the standard deviation (0.5 SD) [26]. The 0.5 SD of the FFI and AOFAS were 10 and 10.5, respectively. Patients with a POWI score a 17 points difference compared to patients without a POWI, resulting in a worse FFI. This implicates that a clinically relevant difference exists between the two groups. The estimated MCID was not reached in the AOFAS hindfoot score. Even though patients scored higher on all items of the EQ-5D, the occurrence of a POWI was not statistically associated with worse health-related QOL.

The VAS on overall patient satisfaction was significantly higher in patients without POWI (*p* = 0.01). Additional admissions, a prolonged hospital stay, additional surgical procedures and costs of wound dressings could all contribute to this inconvenience.

Importantly, almost one-third of patients required adjustment of work environment following calcaneal fracture surgery. This emphasizes the impact a calcaneal fracture has on day-to-day life, and supports the statement that a calcaneal fracture is a life-changing event [27].

We found a high rate of implant removal of 52.1 %. This is in concordance with the literature [28]. Patients without implant removal scored higher in the FFI and therefore show worse functional outcome as opposed to a previous study [14]. Patients with implant removal scored better with a median score of 17 versus 8. This might be a result of implant removal because of symptoms (e.g. pain, palpable screws, stiffness), which are reported in about three quarters of patients [29]. This is in line with previous literature with 79 % of patients reporting less complaints as a result of implant removal following calcaneal fracture surgery [29].

Of interest, we showed that a secondary fusion is indicated more frequently following POWI. This might be explained by additional joint damage caused by infection [30]. In addition, patients reported on ankle stiffness more frequently following POWI, which further contributes to this hypothesis. Patients with secondary arthrodesis scored worse on all outcome scores.

To the best of our knowledge, only three studies have been performed on foot and ankle surgery, with a main focus on postoperative wound complications and outcome effects [14, 31, 32]. In the study by de Groot et al., a retrospective analysis was performed on outcome in 39 patients with an intra-articular calcaneal fracture. They revealed no significant difference between patients with and without a wound complication [14]. However, two-thirds of the reported complications were wound dehiscences, which were not included in the current study. Korim et al. [31] found that both deep and superficial infections result in lower functional outcome scores in a case–control study following operative fixation of fractures of the ankle. Schepers et al. [32] investigated the effect of delay in surgery in closed ankle fractures on occurrence of POWI. Delay in surgery was associated with a significant increase in wound complications, resulting in a lower functional outcome at follow-up of almost 4 years. Delay of definite fixation of closed, intra-articular calcaneal fractures did not decrease wound complication rates when using the extensile lateral approach and an increased wound complication rate when using less invasive approaches was found [10].

The current study is mainly limited by its retrospective character. Even though we received an above average response rate of 70 %, we were unable to locate a considerable percentage of patients. Our non-attenders showed no differences in incidence of POWI, but were more often male and younger, which is similar to a study of Murnaghan and Buckley [33]. This most likely did not affect our results, because no association was found between these characteristics and outcome in the univariate analysis.

In conclusion, our results implicate that postoperative wound infection leads lower functional outcome scores following calcaneal fracture surgery, but no statistical significance was reached. In addition, patients do not report significant worse QOL or physical impairment. Overall patient satisfaction was significantly lower in case of a postoperative wound infection.
